# Supplementary material for: Prompt HIV diagnosis and antiretroviral treatment in postpartum women is crucial for prevention of mother to child transmission during breastfeeding: Survey results in a high HIV prevalence community in southern Mozambique after the implementation of Option B+
Source: PLoS One. 2022 Aug 2;17(8):e0269835. doi: 10.1371/journal.pone.0269835 (PMC9345360; doi:10.1371/journal.pone.0269835)
Supplement: S1 Appendix — (ZIP) [file pone.0269835.s001.zip › SSP_METRO_001_A02b_v01_EN.pdf]

<table>
<tr><td colspan="2">SSP_METRO_001_A02b_v01_EN</td><td colspan="2">Field Recruitment visit</td></tr>
<tr><td>cism centro de investigação em saúde de manhiça</td><td>Study: METRO
Survey: CRF Identification</td><td></td><td>Serial Number</td></tr>
</table>

<table>
<tr><th></th><th>CHILD Information</th></tr>
<tr><td>1.</td><td>CHILD Perm_id |__|__|__|__|__| - |__|__|__|__| - |__|__|</td></tr>
<tr><td>2.</td><td>CHILD Check number |__|</td></tr>
<tr><td>3.</td><td>CHILD Study number METR - |__|__|__|__|__|</td></tr>
<tr><td>4.</td><td>House found 1= Yes 2= No
4.1 If YES, current house number |__|__|__|__|__|-|__|__|__|__|
4.2 If NO, why? 1= House not found 2= Destroy house 3= Abandon house
4= Other |__|__|__|__|__|__|__|__|__|</td></tr>
<tr><td>5.</td><td>CHILD found 1= Yes 2= No
5.1 If NO, why? 1 = Ausent 2= Dead 3= Emigrant 4= Other |__|__|__|__|__|__|__|__|__|__|__|__|
("Ausent" just if the participant was visited 3 times)
5.2 If 5.1 is "dead", age at death |__|__| 1= Days 2= Months 3= years
5.3 If 5.1 is "dead", date of death |__|__|- |__|__|__|__|-201|__| 1= Don`t know</td></tr>
<tr><th></th><th>MOTHER Information</th></tr>
<tr><td>6.</td><td>MOTHER Perm_id |__|__|__|__|__| - |__|__|__|__| - |__|__| 1= Don`t have</td></tr>
<tr><td>7.</td><td>MOTHER Check number |__|</td></tr>
<tr><td>8.</td><td>MOTHER Study number METR - |__|__|__|__|__|</td></tr>
<tr><td>9.</td><td><u>Biological</u> MOTHER found 1= Yes 2= No
9.1 If NO, why? 1 = Ausent 2= Dead 3= Emigrant 4= Other |__|__|__|__|__|__|__|__|__|__|__|__|
("Ausent" just if the participant was visited 3 times)
9.2 If 9.1 is "dead", age at death |__|__|
9.3 If 9.1 is "dead", date of death |__|__|- |__|__|__|__|-201|__| 1= Don`t know</td></tr>
<tr><td>10.</td><td>If 9 is YES, did the MOTHER sign the informed consent?
1 = Yes 2= No 3= Just for her 4= Just for the child
10.1 If NO, why? 1 = Refusal 2= Partner refusal 3= Other |__|__|__|__|__|__|__|__|__|__|__|__|
If the participant refuse been visited and he/she didn`t sign the informed consent, finish here the interview</td></tr>
<tr><td>11.</td><td>If 9 is NO, who is the caregiver of the child?
1= Father
2= Grandmother
3= Sister
4= Brother
5= Aunt
6= Uncle
7= Mother in law
8= Sister in law
9= Don`t know
10=Refusal
11=Other |__|__|__|__|__|__|__|__|__|__|__|__|</td></tr>
<tr><th></th><th>CAREGIVER Information</th></tr>
<tr><td>12.</td><td>If question 9 is NO, CAREGIVER perm_id |__|__|__|__|__| - |__|__|__|__| - |__|__| 1= Don`t have</td></tr>
<tr><td>13.</td><td>CAREGIVER Check number |__|</td></tr>
<tr><td>14.</td><td>CAREGIVER Study number METR - |__|__|__|__|__|</td></tr>
<tr><td>15.</td><td>CAREGIVER found 1 = Yes 2= No
15.1 If NO, why? 1 = Ausent 2= Dead 3= Emigrant 4= Other __|__|__|__|__|__|__|__|__|__|__|__|</td></tr>
</table>

	("Ausent" just if the participant was visited 3 times)
16.	**If 15 is YES, did the CAREGIVER sign the informed consent?**<br>1 = Yes 2= No 3= Just for her 4= Just for the child<br>16.1 If NO, why? 1 = Refusal 2= Partner refusal 3= Other |__|__|__|__|__|__|__|__|__|__|__| ***If the participant refuse been visited and he/she didn`t sign the informed consent, finish here the interview***
	FIM
17.	Counselhor |__|__|__|
18.	Date of visit |__|__|- |__|__|__|-201|__|
