# Supplementary material for: Prompt HIV diagnosis and antiretroviral treatment in postpartum women is crucial for prevention of mother to child transmission during breastfeeding: Survey results in a high HIV prevalence community in southern Mozambique after the implementation of Option B+
Source: PLoS One. 2022 Aug 2;17(8):e0269835. doi: 10.1371/journal.pone.0269835 (PMC9345360; doi:10.1371/journal.pone.0269835)
Supplement: S1 Appendix — (ZIP) [file pone.0269835.s001.zip › SSP_METRO_001_A03b_v02_EN.pdf]

cism centro de investigação em saúde de manhiça	**Study: METRO**<br>**Survey: CRF Mother home-based visit**		Serial Number

SOCIO-DEMOGRAPHICS INFORMATION	
1.	**MOTHER Study Number** **METR - |__|__|__|__|**
2.	**Age in years** |__|__| years
3.	**Current Household** |__|__|__|__| - |__|__|__|
4.	**Does the mother live in the same household than the child?**<br>**1=** Yes<br>**2=** No
5.	**MOTHER Marital status:**<br>**1=** Single<br>**2=** Married<br>**3=** Co-habiting<br>**4=** Divorced<br>**5=** Separated<br>**6=** Widowed
6.	**MOTHER highest school grade passed:**<br>**1=** None<br>**2=** Under 5 grade<br>**3=** 5 ª grade<br>**4=** 7 ª grade<br>**5=** 10 ª grade<br>**6=** 12 ª grade<br>**7=** Primary technician<br>**8=** Basic Technician<br>**9=** Medium Technician<br>**10=** High school<br>**11=** Bachelor<br>**12=** Master<br>**13=** Doctor degree<br>**14=** Other<br>**88=** Don`t know
7.	**What is the main material that the house the mother lives in is built with?**<br>**1=** Cement block<br>**2=** Brick<br>**3=** Wood/zinc<br>**4=** Adobe block<br>**5=** Reeds/Bamboo/Palm tree<br>**6=** Clay<br>**7=** Aluminium/Carbon/Paper/Sack/Bark<br>**8=** Other, especify |__|__|__|__|__|__|__|__|__|__|__|__|
8.	**What is the main source of water used for drinking in the mother's house:**<br>**1=** Bottle water<br>**2=** Piped water in the house<br>**3=** Piped water in the yard<br>**4=** Fountain<br>**5=** Waterwell with manual pump<br>**6=** Waterwell without pump<br>**7=** River water/Lake<br>**8=** Rain water

	**9=** Other |__|__|__|__|__|__|__|__|__|__|__|
9.	**What type of toilet do you use at the child's home?**<br>**1=** Flush toilet<br>**2=** Improved latrine<br>**3=** Improved traditional latrine<br>**4=** Not improved latrine<br>5= Latrine shared with other house<br>**6=** Do not`t have<br>**7=** Other |__|__|__|__|__|__|__|__|__|__|__|
10.	**What is the household's primary source of income?**<br>**1=** Farming<br>**2=** Formal employment<br>**3=** Informal employment<br>**4=** Don't know/refuse to answer<br>**5=** Other |__|__|__|__|__|__|__|__|__|__|__|
11.	**What is the mother's religion?**<br>**1=** Catholic<br>**2=** Protestant/Anglican<br>**3=** Christian<br>**4=** Muslim<br>**5=** Hindu<br>**6=** Zione<br>**7=** Animistic<br>**8=** Envangelist / pentecostal<br>**9=** Atheist<br>**10=** Other (specify) |__|__|__|__|__|__|__|__|__|__|__| **88=** Don`t know<br>**99=** Refuse
	**MOTHER`S HISTORY**
12.	**How many times have you been pregnant in total?** |__|__| times 99= refuse to answer
13.	**How many live children do you have in total?** |__|__| children<br>13.1 Do you want to test those under 48 months? **1=** Yes **2=** No 3= Do not have <48m
14.	**Did you have any children who were born alive but died afterwards?**<br>**1=** Yes **2=** No **3=** Don't know
15.	*Fill for each of the child that died*<br>If 14 is YES, at what age did he/she die |__|__| **1=** Days **2=** Months **3=** Years **4=** Don`t know
16.	**Have you ever been tested for HIV?** **1=** Yes **2=** No **3=** Don`t know
17.	**Where were you tested FIRST for HIV?**<br>**1=** Mozambique<br>**2=** South Africa<br>**3=** Dont`t know<br>**4=** Other |__|__|__|__|__|__|__|__|__|__|__|
18.	**Where were you tested LAST for HIV?**

	**5=** Mozambique<br>**6=** South Africa<br>**7=** Didn`t know<br>**8=** Other |__|__|__|__|__|__|__|__|__|__|__|__|
19.	**When was your LAST HIV test?** |__|__| **1=** Days **2=** Months **3=** Years **4=** Dont`t know
20.	***Ask to see the documentation that shows the last test (ficha prenatal/ or cartão da criança/ or cartão de seguimento do GATV)***<br>**Did the MOTHER shown any documentation?** 1= Yes 2= No
21.	If 20 is YES, which documentation shown?<br>***1=*** ANC card/*caderneta da mulher*<br>***2=*** *Cartão da criança*<br>**3=** *Cartão de seguimento do GATV* (ATS negative result)<br>**4=** *Cartão de seguimento nas consultas de HIV* (NID card)<br>**5=** Other |__|__|__|__|__|__|__|__|__|__|__|__|
22.	If 20 is YES, date of the LAST test in the document |__|__|/|__|__|/|__|__|__|__| 888= Don`t have
23.	If 20 is NO, why?<br>**1=** Don`t have<br>**2=** Lost<br>**3=** Refusal<br>**4=** Not accessible in that moment ***(will show another day)***<br>**5=** Other |__|__|__|__|__|__|__|__|__|__|__|__|
24.	**What was the result of your last HIV test?** 1= Positive 2= Negative 3= Indeterminate 4= Don`t know
25.	**What was the location of your last HIV test?**<br>**1=** Home<br>**2=** Health Unit - VCT<br>**3=** Health unit - PICT<br>**4=** Health Unit – ANC<br>**5=** CCR (PMTCT program)<br>**6=** Maternity<br>**7=** Emergency<br>**8=** Health campaigns<br>**9=** Other, specify |__|__|__|__|__|__|__|__|__|__|__|__| **88=** Don`t know
26.	**Was your LAST HIV test done when you were pregnant?** **1=** Yes **2=** No **3=** Don`t know
27.	**If 26 is YES, was you pregnant with this CHILD?** **1=** Yes **2=** No **3=** Don`t know
28.	**Did you ever receive medication for HIV?**<br>**1=** Yes, in Manhiça District<br>**2=** Yes, elsewhere<br>**3=** No<br>**4=** Don`t know
29.	***Ask to the participant to show the NID card***<br>**If 27 is YES, did the MOTHER shown her NID card?** **1=** Yes **2=** No
30.	If 29 is YES, write the NID<br>**1=** C.S.Manhiça |__|__| /|__|__|__|/|__|__|/|__|__|__|__|__| **2=** Elsewhere |__|__|__|__|__|__|__|__|__|__|__|__|
31.	If 29 is NO, why? **1=** Refusal **2=** Lost **3=**Don`t have **4=** Other |__|__|__|__|__|__|__|__|__|__|

	**CHILD CHARACTERISTICS**
32.	**CHILD age (*fill by the counselhor)***<br>**1=** < 18 months<br>**2=** > 18 months
33.	**From the biological children, what is this child's birth order?**<br>Put the position (1= first, 2= second...) |__|__| **88=** Don`t know
34.	**Was the CHILD born in Mozambique?** **1=** Yes **2=** No **3=** Don`t know
35.	**Where was the child delivered?**<br>**1=** Peripheral Health Facility<br>**2=** Manhiça Distrital Hospital<br>**3=** Home<br>**4=** Traditional Healer`s house<br>**5=** On the way to the health facility<br>**6=** Refusal<br>**88=** Don`t know
36.	**Did this child breastfeed at all in the last two months?** **1=** Yes **2=** No
37.	**If 35 is NO, how long ago did the child stop breastfeeding?**<br>|__|__| **1=** Months **2=** Years **3=** Don`t know
38.	**Was this child ever tested for HIV?** **1=** Yes **2=** No **3=** Don`t know
39.	***Only if question 38 was YES***<br>**At what age was the child tested for HIV for the FIRST time?**<br>**1=** < 2 months<br>**2=** 2 months – 1 year<br>**3=** > 1 year<br>**88=** Don`t know
40.	**How many times was this child tested for HIV?**<br>**1=** Once<br>**2=** Twice<br>**3=** > twice<br>**88=** Don`t know
41.	**What was the result of this child's FIRST/only HIV test?**<br>**1=** Positive<br>**2=** Negative<br>**3=** Indeterminate<br>**88=** Don`t know
42.	**What was the result of this child's LAST HIV test?**<br>**1=** Positive<br>**2=** Negative<br>**3=** Indeterminate<br>**4=** Do not have more tests<br>**88=** Don`t know
43.	**Did the CHILD ever receive medication for HIV?**

	**1=** Yes **2=** No **3=** Don`t know
44.	***Ask the participant to show the " cartão da CRIANÇA"***<br>**Did the MOTHER show a child's health document?** **1=** Yes **2=** No
45.	If 44 is YES, what documents did she show?<br>***1=*** ANC card/*caderneta da mulher*<br>***2=*** *Cartão da criança* (child card)<br>**3=** *Cartão de seguimento do GATV* (ATS negative result)<br>**4=** *Cartão de seguimento nas consultas de HIV* (NID card)<br>**5=** Other |__|__|__|__|__|__|__|__|__|__|__|__|
46.	If 44 is YES, date of the LAST test in the document |__|__|/|__|__|/|__|__|__|__| 1= Don`t have
47.	If 44 is YES, write the CHILD NID number<br>**1=** C.S.Manhiça |__|__| /|__|__|__|/|__|__|/|__|__|__|__|__| **2=** Fora da Manhiça |__|__|__|__|__|__|__|__|__|__|__|__|__|
48.	If 44 is NO , why?<br>**1=** Don`t have<br>**2=** Lost<br>**3=** Refusal<br>**4=** Not accessible in that moment ***(turn in another day)***<br>**5=** Other |__|__|__|__|__|__|__|__|__|__|__|__|
	**TEST RESULTS**
49.	***Offer HIV test for MOTHER and CHILD***<br>**Mother tested for HIV in household:**<br>**1=** Yes<br>**2=** No
50.	**If 49 is YES, MOTHER HIV result:**<br>**1=** Positive<br>**2=** Negative<br>**3=** Indeterminate
51.	**If 49 is NO, why?**<br>**1=** Known positive on ART (NID document shown/ePTS)<br>**2=** No, known negative previous 2 months (document shown)<br>**3=** Mother deceased<br>**4=** Refusal<br>**5=** Partner refusal<br>**6=** Other |__|__|__|__|__|__|__|__|__|__|__|__|
52.	**MOTHER sample (DBS) collected?** **1=** Yes **2=** No **3=** Not aplicable
53.	**NIDA MOTHER** [ ]
54.	**If the CHILD is > 18 m, tested for HIV in household:**<br>**1=** Yes<br>**2=** No
55.	**If 54 is YES, HIV result:**

	**1=** Positive<br>**2=** Negative<br>**3=** Indeterminate
56.	**If 53 is NO, why?**<br>**1=** Known positive on ART (NID document shown/ePTS)<br>**2=** No, known negative previous 2 months (document shown)<br>**3=** mother deceased<br>**4=** Mother refusal<br>**5=** Other |__|__|__|__|__|__|__|__|__|__|__|__|
57.	**CHILD sample (DBS) collected?** **1=** Yes **2=** No **3=** Not aplicable
58.	**NIDA CHILD** ☐
59.	**If the CHILD is < 18 m, DBS sample collected in household:**<br>**1=** Yes<br>**2=** No
60.	**If 59 is NO, why?**<br>**1=** Known positive on ART (NID document shown/ePTS)<br>**2=** Child dead<br>**3=** Mother refusal<br>**4=** Other |__|__|__|__|__|__|__|__|__|__|__|__|
	**PREGNANCY INFORMATION**
61.	**During this child's pregnancy, did you attend antenatal care?**<br>**1=** Yes **2=** No **3=** Don`t know
62.	**Before this child's pregnancy, did the MOTHER know her HIV status?**<br>**1=** Yes **2=** No **3=** Don`t know
63.	***If the MOTHER refere not know her HIV status before this pregnancy***<br>**During this child's pregnancy, were you tested for HIV?**<br>**1=** Yes **2=** No **3=** Don`t know
64.	**During this child's pregnancy, how many times were you tested for HIV?**<br>**1=** Once<br>**2=** Twice<br>**3=** > twice<br>**88=** Don`t know
65.	**During this child's <u>pregnancy</u> or in <u>labor</u>/<u>delivery</u>, what was the result of your FIRST HIV test?**<br>**1=** Positive<br>**2=** Negative<br>**3=** Indeterminate<br>**88=** Don`t know
66.	**During this child's <u>pregnancy</u> or in <u>labor</u>/<u>delivery</u>, what was the result of your only or LAST HIV test?**<br>**1=** Positive<br>**2=** Negative

	**3=** Indeterminate<br>**88=** Don`t know
67.	**During this child's pregnancy, did you receive HIV medications? Or medicine to help prevent the child from getting HIV?**<br>**1=** Yes **2=** No **3=** Don`t know
68.	***Ask the participant to see her card and the CHILD documentation that shows the treatment followed***<br>**What documents did she show? (*Multioptions*)**<br>**1=** ANC card/*caderneta da mulher*<br>**2=** *Cartão de seguimento nas consultas de HIV* (NID card MOTHER)<br>**3=** *Cartão da CRIANÇA* (child card)<br>**4=** *Cartão de seguimento nas consultas de HIV* (NID card CHILD)<br>**5=** Don´t have<br>**6=** Lost<br>**7=** Not accesible in this moment<br>**8=** Refusal<br>**9=** Other |__|__|__|__|__|__|__|__|__|__|__|__|
69.	**What kind of medication did you receive during pregnancy?**<br>**1=** AZT and NVP+ Duovir (during labor and 7 days after)<br>**2=** Option B+ Triple ARV<br>**3=** NVP single dose during the labor<br>**4=** Don't know/ Unable to stablish<br>**5=** Impossible to read in the *cartao* because of the bad conservation<br>**6=** Other |__|__|__|__|__|__|__|__|__|__|__|__|
70.	**After this child was born, did you receive medication?** **1=** Yes **2=** No **3=** Don`t know
71.	**After this child was born, did he/she take any medicine to prevent HIV infection?**<br>**1=** Yes **2=** No **3=** Don`t know
72.	**After this child was born, which medicinedid the CHILD take to prevent HIV infection?**<br>**1=** Nevirapina<br>**2=** AZT<br>**3=** don't know/unable to establish<br>**4=** Impossible to read in the *cartao* because of the bad conservation<br>**5=** Other |__|__|__|__|__|__|__|__|__|__|__|__|
73.	**After this child was born, for how long did he/she take medicine to prevent HIV infection?**<br>|__|__| **1=** Months **2=** Years **3=** Days **4=** Don´t know
74.	Has the child ever received a blood transfusion? **1=** Yes **2=** No **3=** Don`t know
75.	If 74 is YES, how many times |__|__|
76.	If 74 is YES, when was the last time? |__|__| **1=** Months **2=** Years **3=** Don´t know
	**FIM**
77.	Counselor code |__|__|__|
78.	Visit date |__|__|- |__|__|__|-201|__|
