# Supplementary material for: Prompt HIV diagnosis and antiretroviral treatment in postpartum women is crucial for prevention of mother to child transmission during breastfeeding: Survey results in a high HIV prevalence community in southern Mozambique after the implementation of Option B+
Source: PLoS One. 2022 Aug 2;17(8):e0269835. doi: 10.1371/journal.pone.0269835 (PMC9345360; doi:10.1371/journal.pone.0269835)
Supplement: S1 Appendix — (ZIP) [file pone.0269835.s001.zip › SSP_METRO_001_A04b_v02_EN.pdf]

SSP_METRO_001_A04b_v02_EN		Field Recruitment Visit	
cism centro de investigação em saúde de manhiça	**Study: METRO**<br>**Survey: CRF Visit with CAREGIVER**		Serial Number

	SOCIO-DEMOGRAPHICS INFORMATION
1.	**CAREGIVER Study Number** **METR - |__|__|__|__|**
2.	**CAREGIVER Age in years** |__|__| years
3.	**Current Household of the CAREGIVER** |__|__|__|__| - |__|__|__|
4.	**CAREGIVER Gender** **1=** Male **2=** Female
5.	**What is the relationship of the CAREGIVER with the MOTHER?**<br>**1=** Father/ Mother<br>**2=** Grandparent<br>**3=** Siter/Brother<br>**4=** Neighbour<br>**5=** Uncle/Aunt<br>**6=** Bother/sister in law<br>**7=** No relationship<br>**8=** Other |__|__|__|__|__|__|__|__|__|__|__|__|
6.	**What is the relationship of the CAREGIVER with the CHILD?**<br>**1=** Father<br>**2=** Step mother<br>**3=** Grandmother<br>**4=** Sister<br>**5=** Brother<br>**6=** Neighbour<br>**7=** Uncle<br>**8=** Aunt<br>**9=** Godfather<br>**10=** Godmother<br>**11=** Other |__|__|__|__|__|__|__|__|__|__|__|__|
7.	**MOTHER Marital status:**<br>**1=** Single (never lived in a marital situation)<br>**2=** Married<br>**3=** Co-habiting<br>**4=** Divorced<br>**5=** Separated<br>**6=** Widowed
8.	**MOTHER highest school grade passed:**<br>**1=** None<br>**2=** Under 5 grade<br>**3=** 5 ª grade<br>**4=** 7 ª grade<br>**5=** 10 ª grade<br>**6=** 12 ª grade<br>**7=** Primary technician<br>**8=** Basic Technician<br>**9=** Medium Technician<br>**10=** High school<br>**11=** Bachelor<br>**12=** Master<br>**13=** Doctor degree<br>**14=** Other |__|__|__|__|__|__|__|__| **88=** Don`t know
9.	**What is the main material that the house the CHILD sleeps in is built with?**

	**1=** Cement block<br>**2=** Bricks<br>**3=** Wood/zinc<br>**4=** Adobe block<br>**5=** Reeds/Bamboo/Palm tree<br>**6=** Clay<br>**7=** Aluminium/Carbon/Paper/Sack/Bark<br>**8=** Other, especify |__|__|__|__|__|__|__|__|__|__|__|
10.	**What is the main source of water used for drinking in the CHILD's house:**<br>**1=** Bottle water<br>**2=** Piped water in the house<br>**3=** Piped water in the yard<br>**4=** Fountain<br>**5=** Waterwell with manual pump<br>**6=** Waterwell without pump<br>**7=** River water/Lake<br>**8=** Rain water<br>**9=** Other |__|__|__|__|__|__|__|__|__|__|__|
11.	**What type of toilet do you use at the CHILD's home?**<br>**1=** Flush toilet<br>**2=** Improved latrine<br>**3=** Improved traditional latrine<br>**4=** Not improved latrine<br>5= Latrine share with other house<br>**6=** Do not have<br>**7=** Other |__|__|__|__|__|__|__|__|__|__|__|
12.	**What is the CHILD household's primary source of income?**<br>**1=** Farming<br>**2=** Formal employment<br>**3=** Informal employment<br>**4=** Don't know/refuse to answer<br>**5=** Other |__|__|__|__|__|__|__|__|__|__|__|
13.	**What is the MOTHER´s religion?**<br>**1=** Catholic<br>**2=** Protestant/Anglican<br>**3=** Christian<br>**4=** Muslim<br>**5=** Hindu<br>**6=** Zione<br>**7=** Animistic<br>**8=** Envangelist / pentecostal<br>**9=** Atheist<br>**10=** Other (especify) |__|__|__|__|__|__|__|__|__|__|__| **88=** Don`t know<br>**99=** Refuse
	**MOTHER`S HISTORY**

<table>
<tr><td>14.</td><td>Did the child’s mother have other children? 1= Yes 2= No 3= Don`t know 4= Refusal</td></tr>
<tr><td>15.</td><td>How many children did she have in total? |__|__| children
15.1 Do you want to test those under 48 months? 1= Yes 2= No 3= Do not have <48m</td></tr>
<tr><td>16.</td><td>Did she have any children that were born alive but died afterwards?
1= Yes 2= No 3= Don`t know</td></tr>
<tr><td>17.</td><td>Fill for each of the child that died
If 16 is YES, at what age did he/she die |__|__| 1= Days 2= Months 3= Years 4= Don`t know</td></tr>
<tr><td>18.</td><td>To your knowledge was this child’s mother ever tested for HIV? 1= Yes 2= No 3= Don`t know</td></tr>
<tr><td>19.</td><td>Ask to see the documentation that shows the last test of the MOTHER (ficha prenatal/ or cartão da criança/ or cartão de seguimento do GATV)
Did the CAREGIVER show any documentation? 1= Yes 2= No</td></tr>
<tr><td>20.</td><td>If 19 is YES, which documentation shown? (Multiple choices)
1= ANC card/caderneta da mulher
2= Cartão da criança
3= Cartão de seguimento do GATV (ATS negative result)
4= Cartão de seguimento nas consultas de HIV (NID card)
5= Other |__|__|__|__|__|__|__|__|__|__|__|__|__|__|</td></tr>
<tr><td>21.</td><td>If 19 is YES, date of the LAST test in the document |__|__|/|__|__|/|__|__|__|__| 888= Don`t have</td></tr>
<tr><td>22.</td><td>If the CAREGIVER shows the NID card, write the NID
1= C.S.Manhiça |__|__| /|__|__|__|/|__|__|/|__|__|__|__|__|
2= Elsewhere |__|__|__|__|__|__|__|__|__|__|__|__|__|__|</td></tr>
<tr><td>23.</td><td>If 19 is NO, why?
1= Don`t have
2= Lost
3= Refusal
4= Not accessible in that moment (will show another day)
5= Other |__|__|__|__|__|__|__|__|__|__|__|__|__|__|</td></tr>
<tr><td>24.</td><td>If 19 is NO, to your knowledge when was her last HIV test?
|__|__| 1= Months 2= Years 3= Don`t know</td></tr>
<tr><td>25.</td><td>To your knowledge was the mother of this child HIV positive when she was pregnant?
1= Yes 2= No 3= Don`t know</td></tr>
<tr><td>26.</td><td>To your knowledge was the mother of this child diagnosed with HIV after this child was born?
1= Yes 2= No 3= Don`t know</td></tr>
<tr><td>27.</td><td>To your knowledge, when was the mother of this child diagnosed with HIV?
|__|__| 1= Months 2= Years 3= Don`t know</td></tr>
<tr><td></td><td>CHILD CHARACTERISTICS</td></tr>
<tr><td>28.</td><td>Child age (filled by the counselhor)
1= < 18 months
2= > 18 months</td></tr>
<tr><td>29.</td><td>From the biological children, what is this child’s birth order?
Put the position (1= first, 2= second...) |__|__| 88= Don`t know</td></tr>
<tr><td>30.</td><td>Was the CHILD born in Mozambique? 1= Yes 2= No 3= Don`t know</td></tr>
<tr><td>31.</td><td>Where was the child delivered?
1= Peripheral Health Facility
2= Manhiça Distrital Hospital</td></tr>
</table>

	**3=** Home<br>**4=** Traditional Healer`s house<br>**5=** On the way to the health facility<br>**6=** Refusal<br>**88=** Don`t know
32.	**Did this child breastfeed at all in <u>the last two months</u>?** **1=** Yes **2=** No
33.	**Who breastfeed the child?**<br>**1=** The mother<br>**2=** The caregiver<br>**3=** Other (establish the relationship with the child) |__|__|__|__|__|__|__|__|__|__|__|
34.	**If 32 is NO, how long ago did the child stop breastfeeding?**<br>|__|__| **1=** Months **2=** Years **3=** Don`t know
35.	**Was this child ever tested for HIV?** **1=** Yes **2=** No **3=** Don`t know
36.	***Only if question 35 was YES***<br>**At what age was the child tested for HIV for the FIRST time?**<br>**1=** < 2 months<br>**2=** 2 months – 1 year<br>**3=** > 1 year<br>**88=** Don`t know
37.	**How many times was this child tested for HIV?**<br>**1=** Once<br>**2=** Twice<br>**3=** > twice<br>**88=** Don`t know
38.	**What was the result of this child's FIRST/only HIV test?**<br>**1=** Positive<br>**2=** Negative<br>**3=** Indeterminate<br>**88=** Don`t know
39.	**What was the result of this child's LAST HIV test?**<br>**1=** Positive<br>**2=** Negative<br>**3=** Indeterminate<br>**4=** Do not have any other test done<br>**88=** Don`t know
40.	**Did the CHILD ever receive medication for HIV?**<br>**1=** Yes **2=** No **3=** Don`t know
41.	***Ask the participant to show the " cartão da CRIANÇA"***<br>**Did the CAREGIVER show a child's health document?** **1=** Yes **2=** No
42.	If 41is YES, what documents did she shown?<br>***1=*** ANC card/*caderneta da mulher*<br>***2=*** *Cartão da criança*<br>**3=** *Cartão de seguimento do GATV* (ATS negative result)<br>**4=** *Cartão de seguimento nas consultas de HIV* (NID card)<br>**5=** Other |__|__|__|__|__|__|__|__|__|__|__|__|

43.	If 41 is YES, date of the LAST test in the document |__|__|/|__|__|/|__|__|__|__| 1= Don`t have
44.	If 41 is YES, write the CHILD NID number<br>**1=** C.S.Manhiça |__|__| /|__|__|__|/|__|__|/|__|__|__|__|__| **2=** Fora da Manhiça |__|__|__|__|__|__|__|__|__|__|__|__|__|
45.	If 41 is NO , why?<br>**1=** Don`t have<br>**2=** Lost<br>**3=** Refusal<br>**4=** Not accessible in that moment ***(turn in another day)***<br>**5=** Other |__|__|__|__|__|__|__|__|__|__|__|__|
	**RESULTADOS DO TESTE**
46.	***Offer HIV test for CAREGIVER and CHILD***<br>**If the CHILD is > 18 m, tested for HIV in household:**<br>**1=** Yes<br>**2=** No
47.	**If 46 is YES, HIV result:**<br>**1=** Positive<br>**2=** Negative<br>**3=** Indeterminate
48.	**If 46 is NO, why?**<br>**1=** Known positive on ART (NID document shown/ePTS)<br>**2=** No, known negative previous 2 months (document shown)<br>**3=** Child deceased<br>**4=** Caregiver refusal<br>**5=** Other |__|__|__|__|__|__|__|__|__|__|__|__|
49.	**CHILD sample (DBS) collected?** **1=** Yes **2=** No **3=** Not aplicable
50.	**CHILD NIDA** ☐
51.	**If the CHILD is < 18 m, DBS sample collected in household:**<br>**1=** Yes<br>**2=** No
52.	**If 51 is NO, why?**<br>**1=** Known positive on ART (NID document shown/ePTS)<br>**2=** Child deceased<br>**3=** Caregiver refusal<br>**4=** Other |__|__|__|__|__|__|__|__|__|__|__|__|
	**PREGNANCY INFORMATION**
53.	**During this child's pregnancy, did the MOTHER attend antenatal care?**<br>**1=** Yes **2=** No **3=** Don`t know
54.	**Before this child's pregnancy, did the MOTHER known her HIV status?**<br>**1=** Yes **2=** No **3=** Don`t know
55.	***If the CAREGIVER refere not know the MOTHER HIV status before the pregnancy***<br>**During this child's pregnancy, were the MOTHER tested for HIV?**<br>**1=** Yes **2=** No **3=** Don`t know

56.	**During this child's pregnancy, how many times was the MOTHER tested for HIV?**<br>**1=** Once<br>**2=** Twice<br>**3=** > twice<br>**88=** Don`t know
57.	**During this child's <u>pregnancy</u> or in <u>labor</u>/<u>delivery</u>, what was the result of the mother FIRST HIV test?**<br>**1=** Positive<br>**2=** Negative<br>**3=** Indeterminate<br>**88=** Don`t know
58.	**During this child's <u>pregnancy</u> or in <u>labor</u>/<u>delivery</u>, what was the result of the MOTHER only or LAST HIV test?**<br>**1=** Positive<br>**2=** Negative<br>**3=** Indeterminate<br>**88=** Don`t know
59.	**During this child's <u>pregnancy</u>, did the mother receive medication for HIV? Or medication to prevent the transmission of the virus to the newborn?**<br>**1=** Yes **2=** No **3=** Don`t know
60.	***Ask the participant to see the MOTHER card and the CHILD documentation that shows the treatment followed***<br>**What documents did the CAREGIVER shown? (*Multioptions*)**<br>**1=** ANC card/*caderneta da mulher*<br>**2=** *Cartão de seguimento nas consultas de HIV* (NID card MOTHER)<br>***3=*** *Cartão da CRIANÇA*<br>**4=** *Cartão de seguimento nas consultas de HIV* (NID card CHILD)<br>**5=** Don´t have<br>**6=** Lost<br>**7=** Not accessible in that moment ***(turn in another day)***<br>**8=** Refusal<br>**9=** Other |__|__|__|__|__|__|__|__|__|__|__|__|__|
61.	**What kind of medication did the MOTHER receive during pregnancy?**<br>**1=** AZT and NVP+ Duovir (during labor and 7 days after)<br>**2=** Option B+ Triple ARV<br>**3=** NVP single dose during the labor<br>**4=** Don't know/ Unable to stablish<br>**5=** Impossible to read in the *cartao* because of the bad conservation<br>**6=** Other |__|__|__|__|__|__|__|__|__|__|__|__|__|
62.	**After this child was born, did the MOTHER receive medication?**<br>**1=** Yes **2=** No **3=** Don`t know
63.	**After this child was born, did he/she take any medicine to prevent HIV infection?**<br>**1=** Yes **2=** No **3=** Don`t know
64.	**After this child was born, which medicine did the CHILD take to prevent HIV infection?**<br>**1=** Nevirapina<br>**2=** AZT<br>**3=** Don't know/unable to establish<br>**4=** Impossible to read in the *cartao* because of the bad conservation

	**5=** Other |__|__|__|__|__|__|__|__|__|__|__|__|
65.	**After this child was born, for how long did he/she take medicine to prevent HIV infection?**<br>|__|__| **1=** Months **2=** Years **3=** Don´t know
66.	**Has the child ever received a blood transfusion?** **1=** Yes **2=** No **3=** Don`t know
67.	**If 66 is YES, how many times** |__|__|
68.	**If 66 is YES, when was the last time?** |__|__| **1=** Months **2=** Years **3=** Don´t know
	**FIM**
69.	Counselhor code |__|__|__|
70.	Visit date |__|__|- |__|__|__|-201|__|
