# Supplementary material for: Prompt HIV diagnosis and antiretroviral treatment in postpartum women is crucial for prevention of mother to child transmission during breastfeeding: Survey results in a high HIV prevalence community in southern Mozambique after the implementation of Option B+
Source: PLoS One. 2022 Aug 2;17(8):e0269835. doi: 10.1371/journal.pone.0269835 (PMC9345360; doi:10.1371/journal.pone.0269835)
Supplement: S1 Appendix — (ZIP) [file pone.0269835.s001.zip › SSP_METRO_001_A05b_v01_EN.pdf]

Field Recruitment visit

cism centro de investigação em saúde de manhiça	**Study: METRO**<br>**Survey: CRF Children testing**		Serial Number

	**OTHER CHILD FROM THE HOUSEHOLD RESULTS**
1.	**MOTHER/CAREGIVER Study Number** **METR - |__|__|__|__|**
2.	**How many children under 48 months live in the house?** |__|__|
3.	**How many were tested?** |__|__|
4.	***Repeat for each of the children NOT tested***<br>**Why the child was not tested?**<br>1= Mother/caregiver refusal<br>2= Familiar refusal<br>3= Child not found<br>**4=** Known positive on ART (NID document shown/ePTS)<br>**5=** No, known negative previous 2 months (document shown)<br>6= Other |__|__|__|__|
5.	***Repeat for each of the children >18 months tested***<br>**Age in months** |__|__| months
6.	**What was the result of the test?**<br>**1=** Positive<br>**2=** Negative<br>**3= Indeterminate**
7.	***Repeat for each of the children <18 months tested***<br>**CHILD-|__|__|__|__|**
8.	**CHILD Perm Id** |__|__|__|__| - |__|__|__| - |__|__| 88= Do not have
9.	**CHILD name** **|__|__|__|__|**
10.	**Age in months** |__|__| months
11.	**CHILD gender** **1= men** **2= women**
12.	**CHILD sample (DBS) collected?** **1=** Yes **2=** No **3=** Not aplicable
13.	**NIDA CHILD**
	**FIM**
14.	Counselhor code |__|__|__|
15.	Visit date |__|__|- |__|__|__|-201|__|
