# Supplementary material for: Prompt HIV diagnosis and antiretroviral treatment in postpartum women is crucial for prevention of mother to child transmission during breastfeeding: Survey results in a high HIV prevalence community in southern Mozambique after the implementation of Option B+
Source: PLoS One. 2022 Aug 2;17(8):e0269835. doi: 10.1371/journal.pone.0269835 (PMC9345360; doi:10.1371/journal.pone.0269835)
Supplement: S2 Appendix — (ZIP) [file pone.0269835.s002.zip › SSP_METRO_001_A02_v01_PT.pdf]

<table>
<tr><td>SSP_METRO_001_A02_v01_PT</td><td colspan="3">Visita de recrutamento no campo</td></tr>
<tr><td>cism centro de investigação em saúde de manhiça</td><td>Estudo: METRO<br>Inquérito: CRF identificação</td><td></td><td>Serial Number</td></tr>
</table>

<table>
<tr><td></td><td>Informação da CRIANÇA</td></tr>
<tr><td>1.</td><td>Perm_id CRIANÇA |__|__|__|__| - |__|__|__| - |__|__|</td></tr>
<tr><td>2.</td><td>Digito check CRIANÇA |__|</td></tr>
<tr><td>3.</td><td>Número de estudo da CRIANÇA METR - |__|__|__|__|</td></tr>
<tr><td>4.</td><td>Agregado localizado? 1= Sim 2= Não<br>4.1 Se sim, agregado actual |__|__|__|__|-|__|__|__|<br>4.2 Se não, por quê? 1= Emigraram todos para fora da area 2= Casa destruida<br>3= Ninguem sabe deles 4= Outro |__|__|__|__|__|__|__|</td></tr>
<tr><td>5.</td><td>CRIANÇA localizada 1= Sim 2= Não<br>5.1 Se não, por quê? 1= Ausente 2= Óbito 3= Emigração fora da area 4= Outro |__|__|__|__|__|__|<br>( só preencher “Ausente” se o participante já foi visitado 3 vezes e não foi encontrado)<br>5.2 Se 5.1 é “óbito”, idade da criança no falecimento |__|__| 1= Dias 2= Meses 3= Anos<br>5.3 Se 5.1 é “óbito”, data de falecimento da criança |__|__|- |__|__|__|-201|__| 1= Não sabe responder</td></tr>
<tr><td></td><td>Informação da MÃE</td></tr>
<tr><td>6.</td><td>Perm_id MÃE |__|__|__|__| - |__|__|__| - |__|__| 1= Não tem</td></tr>
<tr><td>7.</td><td>Digito check MÃE |__|</td></tr>
<tr><td>8.</td><td>Número de estudo da MÃE METR - |__|__|__|__|</td></tr>
<tr><td>9.</td><td>MÃE <u>BIOLOGICA</u> localizada 1= Sim 2= Não<br>9.1 Se não, por quê? 1= Ausente 2= Óbito 3= Emigração 4= Outro |__|__|__|__|__|__|__|__|__|<br>( só preencher “Ausente” se a participante já foi visitada 3 vezes)<br>9.2 Se 9.1 é “óbito”, idade da mãe no falecimento |__|__|<br>9.3 Se 9.1 é “óbito”, data de falecimento da mãe |__|__|- |__|__|__|-201|__| 1= Não sabe responder</td></tr>
<tr><td>10.</td><td>Se 9 é SIM, a MÃE assinou consentimento informado?<br>1= Sim 2= Não 3= Só para ela 4= Só para criança<br>10.1 Se não, por quê? 1= Recusa 2= Recusa do parceiro 3= Outro |__|__|__|__|__|__|__|__|__|__|<br>Se a mae não quer participar e recusa assinar o consentimento, terminar aqui o inquérito</td></tr>
<tr><td>11.</td><td>Se 9 é não, qual é a relação do cuidador com a criança?<br>1= Pai<br>2= Avô (masculino)<br>3= Avó (feminino)<br>4= Irmã<br>5= Irmão<br>6= Tia<br>7= Tio<br>8= Madrasta<br>9= Mulher do Irmão<br>10= Recusa responder<br>11= Outro |__|__|__|__|__|__|__|__|__|__|__|</td></tr>
<tr><td></td><td>Informação do CUIDADOR</td></tr>
<tr><td>12.</td><td>Se pergunta 9 é NÃO, perm_id CUIDADOR |__|__|__|__| - |__|__|__| - |__|__| 1= Não tem</td></tr>
<tr><td>13.</td><td>Digito check CUIDADOR |__|</td></tr>
<tr><td>14.</td><td>Número de estudo da CUIDADOR METR - |__|__|__|__|</td></tr>
<tr><td>15.</td><td>CUIDADOR localizado 1= Sim 2= Não<br>15.1 Se não, por quê? 1= Ausente 2= Óbito 3= Emigração 4= Outro |__|__|__|__|__|__|__|__|__|<br>( só preencher “Ausente” se o participante já foi visitado 3 vezes)</td></tr>
</table>

<table>
<tr><td>16.</td><td>Se 15 é SIM, o cuidador assinou consentimento informado?<br>1= Sim 2= Não 3= Só para ela 4= Só para criança<br>16.1 Se não, por quê? 1= Recusa 2= Recusa do parceiro 3= Outro |__|__|__|__|__|__|__|__|__|__|__|<br>Se o participante não quer participar e recusa assinar o consentimento, terminar aqui o inquérito</td></tr>
<tr><td></td><td>FIM</td></tr>
<tr><td>17.</td><td>Código conselheiro |__|__|__|</td></tr>
<tr><td>18.</td><td>Data da visita |__|__|- |__|__|__|-201|__|</td></tr>
</table>
