# Supplementary material for: Prompt HIV diagnosis and antiretroviral treatment in postpartum women is crucial for prevention of mother to child transmission during breastfeeding: Survey results in a high HIV prevalence community in southern Mozambique after the implementation of Option B+
Source: PLoS One. 2022 Aug 2;17(8):e0269835. doi: 10.1371/journal.pone.0269835 (PMC9345360; doi:10.1371/journal.pone.0269835)
Supplement: S2 Appendix — (ZIP) [file pone.0269835.s002.zip › SSP_METRO_001_A03_v02_PT.pdf]

	INFORMAÇÃO SOCIO-DEMOGRÁFICA	
1.	**Número de estudo da MÃE** **METR - |__|__|__|__|**	
2.	**Idade da MÃE em anos** |__|__| anos	
3.	**Agregado actual da MÃE** |__|__|__|__| - |__|__|__|	
4.	**A mãe vive (ou vivia) no mesmo agregado com a criança do estudo?**<br>1 = Sim<br>2 = Nao	
5.	**Estado civil da MÃE:**<br>**1=** Solteira (nunca viveu maritalmente)<br>**2=** Casada<br>**3=** União-de-facto<br>**4=** Divorciada<br>**5=** Separada<br>**6=** Viuva	
6.	**Nível de escolaridade da MÃE:**<br>**1=** Analfabeto<br>**2=** Abaixo de 5 grau<br>**3=** 5 ª classe<br>**4=** 7 ª classe<br>**5=** 10 ª classe<br>**6=** 12 ª classe<br>**7=** Ensino técnico elementar	**8=** Ensino técnico básico<br>**9=** Ensino técnico médio<br>**10=** Bacharel<br>**11=** Licenciatura<br>**12=** Mestrado<br>**13=** Doutoramento<br>**14=** Outro<br>**88=** Não sabe
7.	**Material Principal de construção da casa onde está a dormir a MÃE?**<br>**1=** Blocos de cimento<br>**2=** Bloco de tijolo<br>**3=** Madeira/zinco<br>**4=** Bloco de adobe<br>**5=** Caniço/Pau/Bambu/Palmeira<br>**6=** Pau maticado<br>**7=** Lata/Carvão/Papel/Saco/Casca<br>**8=** Outros, especifique |__|__|__|__|__|__|__|__|__|__|__|	
8.	**Principal fonte de agua de para consumo da MÃE:**<br>**1=** Agua emgarrafada<br>**2=** Agua canalizada dentro de casa<br>**3=** Água canalizada no quintal<br>**4=** Fontanário<br>**5=** Poço/ Furo protegido com bomba manual<br>**6=** Poço sem bomba<br>**7=** Água do Rio/Lago/Lagoa<br>**8=** Água da chuva<br>**9=** Outro |__|__|__|__|__|__|__|__|__|__|__|	

<table>
<tr><td>9.</td><td>Tipo de casa de banho no agregado onde dorme a MAE
1= Retrete ligada a fossa séptica
2= Latrina Melhorada
3= Latrina tradicional melhorada
4= Latrina não melhorada
5= Latrina compartida com um outro agregado
6= Não tem
7= Outro |__|__|__|__|__|__|__|__|__|__|__|__|</td></tr>
<tr><td>10.</td><td>Qual é a principal fonte de rendimento do agregado onde vive a MAE?
1= Camponês
2= Assalariado
3= Sem salario fixo
4= Não sabe ou não quere responder
5= Outro |__|__|__|__|__|__|__|__|__|__|__|__|</td></tr>
<tr><td>11.</td><td>Religião da MÃE:
1= Católica
2= Protestante/Anglicana
3= Cristão indeterminado
4= Islâmica
5= Hindus
6= Zione/ Sião
7= Animistas
8= Envangelica / pentecostal
9= Ateus
10= Outro (especifique) |__|__|__|__|__|__|__|__|__|__|__|__|
88= Não sabe
99= Recusa</td></tr>
<tr><td></td><td>HISTORIA CLINICA DA MÃE</td></tr>
<tr><td>12.</td><td>Quantas vezes ficou grávida em toda a sua vida? |__|__| vezes 99 = Recusa</td></tr>
<tr><td>13.</td><td>Quantas crianças nasceram vivas em total? |__|__| crianças
13.1 Queres testar ao resto das crianças menores de 48m? 1= Sim 2= Não 3= Não tem <48m</td></tr>
<tr><td>14.</td><td>Alguma das crianças que nasceram vivas, morreram depois? 1= Sim 2= Não 3= Não sabe</td></tr>
<tr><td>15.</td><td>Preencher para cada uma das crianças mortas
Se 14 e SIM, idade da criança quando morreu |__|__| 1= Dias 2= Meses 3= Anos 4= Não sabe</td></tr>
<tr><td>16.</td><td>Alguma vez a MÃE fez teste de HIV? 1= Sim 2= Não 3= Não sabe</td></tr>
<tr><td>17.</td><td>A onde é que a MÃE fez o PRIMEIRO teste:
1= Moçambique
2= Africa do Sul
3= Não sabe
4= Outro |__|__|__|__|__|__|__|__|__|__|__|__|</td></tr>
<tr><td>18.</td><td>Em que país a MÃE refere ter feito o ÚLTIMO teste:
1= Moçambique
2= Africa do Sul
3= Não sabe
4= Outro |__|__|__|__|__|__|__|__|__|__|__|__|</td></tr>
</table>

19.	**A quanto tempo a mãe fez o ÚLTIMO teste? |__|__|** 1= Dias 2= Meses 3= Anos 4= Não sabe
20.	***Pedir ao participante para mostrar algum documento que certifique o teste (ficha prenatal/ ou cartão da criança/ ou cartão de seguimento do TARV)***<br>**A MÃE mostrou algum documento?** 1= Sim 2= Não
21.	Se 20 é SIM, que documento apresentou? ***(Nota: Esta pergunta aceita multiplas opçoes)***<br>**1=** Ficha prenatal/caderneta da mulher<br>**2=** Cartão da criança<br>**3=** Cartão de seguimento do GATV<br>**4=** Cartão de seguimento nas consultas de HIV<br>**5=** Outro |__|__|__|__|__|__|__|__|__|__|__|__|
22.	Se 20 é SIM, data do ÚLTIMO teste registado no documento |__|__|/|__|__|/|__|__|__|__| 888= Não tem data
23.	Se 20 é NÃO, porquê?<br>**1=** Não tem<br>**2=** Perdeu<br>**3=** Recusa<br>**4=** Inacessivel **(*voltar noutro dia)***<br>**5=** Outro |__|__|__|__|__|__|__|__|__|__|__|__|
24.	**Qual foi o resultado do ÚLTIMO teste?** 1= Positivo 2= Negativo 3= Indeterminado 4= Não sabe
25.	**A onde é que a MÃE fez o último teste?**<br>**1=** Casa<br>**2=** GATV/SAAJ<br>**3=** Triagem/contentores<br>**4=** CPN<br>**5=** CCR<br>**6=** Maternidade<br>**7=** B.socorro<br>**8=** Feira de Saúde<br>**9=** Outro, especificar |__|__|__|__|__|__|__|__|__|__|__|__| **88=** Não sabe
26.	**A MÃE estava grávida no momento do ÚLTIMO teste?** **1=** Sim **2=** Não **3=** Não sabe
27.	**Se 25 é SIM, estava gravida desta CRIANÇA?** **1=** Sim **2=** Não **3=** Não sabe
28.	**Alguma vez começo a tomar comprimidos contra o HIV?**<br>**1=** Sim, no distrito da Manhiça<br>**2=** Sim, num outro centro de Saude<br>**3=** Não<br>**4=** Não sabe
29.	***Pedir ao participante para mostrar o cartão de seguimento***<br>**Se 27 é SIM, o participante mostrou o cartão de seguimento da MÃE?** **1=** Sim **2=** Não
30.	Se 29 é SIM, escrever o numero do cartão de seguimento da MÃE<br>**1=** C.S.Manhiça |__|__| /|__|__|__|/|__|__|/|__|__|__|__|__| **2=** Fora da Manhiça |__|__|__|__|__|__|__|__|__|__|__|__|__|
31.	Se 29 é NÃO, por quê? 1= Recusa 2= Perdeu 3=Não tem 4.= Inacessivel, 5= Outro<br>|__|__|__|__|__|__|__|__|__|__|
	CARACTERISTICAS DA CRIANÇA
32.	**Idade da CRIANÇA (*preencher pelo conselheiro)***<br>**1=** < 18 meses<br>**2=** > 18 meses
33.	**Das crianças biologicas que teve a MAE, qual é a ordem de nascimento da CRIANÇA?**

	Colocar o numero de ordem (1 = Pimeira; 2= Segunda….) |__|__| 88= Não sabe
34.	**A CRIANÇA foi nascida em Mozambique?** **1=** Sim **2=** Não **3=** Não sabe
35.	**Onde é que a CRIANÇA nasceu?**<br>**1=** Centro de Saúde da periferia<br>**2=** Hospital Distrital da Manhiça<br>**3=** Casa<br>**4=** Na casa do curandeiro<br>**5=** A caminho da unidade sanitaria<br>**6=** Recusa<br>**88=** Não sabe
36.	**A CRIANÇA amamentou nos últimos <u>dois meses</u>?** **1=** Sim **2=** Não
37.	**Se 35 é NÃO, a quanto tempo deixou de amamentar?** |__|__| **1=** Meses **2=** Anos **88=** Não sabe
38.	**Alguma vez a CRIANÇA fez teste de HIV?** **1=** Sim **2=** Não **3=** Não sabe
39.	*O resto das perguntas, só se pergunta 38 for SIM*<br>**A que idade a CRIANÇA foi testada pela PRIMEIRA vez?**<br>**1=** entre 4-8 semanas<br>**2=** 2 meses – 1 ano de idade<br>**3=** > de 1 ano de idade<br>**88=** Não sabe
40.	**Quantas vezes a CRIANÇA fez teste de HIV?**<br>**1=** 1 vezes<br>**2=** 2 vezes<br>**3=** > 2 vezes<br>**88=** Não sabe
41.	**Qual foi o resultado da CRIANÇA no PRIMEIRO/ÚNICO teste?**<br>**1=** Positivo<br>**2=** Negativo<br>**3=** Indeterminado<br>**88=** Não sabe
42.	**Qual foi o resultado da CRIANÇA no ÚLTIMO teste?**<br>**1=** Positivo<br>**2=** Negativo<br>**3=** Indeterminado<br>**4=** Nao tem mais testes feitos<br>**88=** Não sabe
43.	**Alguma vez a CRIANÇA começou a tomar comprimidos/xarope contra o HIV?**<br>**1=** Sim **2=** Não **3=** Não sabe
44.	***Pedir ao participante para mostrar o cartão da CRIANÇA***<br>**A mãe mostrou algum documento da CRIANÇA?** 1= Sim 2= Não
45.	**Se 44 é SIM, que documentação apresentou?**<br>**1=** Ficha prenatal/caderneta da mulher<br>**2=** Cartão da criança<br>**3=** Cartão de seguimento do GATV<br>**4=** Cartão de seguimento da CRIANÇA nas consultas de HIV

	**5=** Outro |__|__|__|__|__|__|__|__|__|__|__|__|__|__|
46.	Se 44 é SIM, data do ÚLTIMO teste registado no documento |__|__|/|__|__|/|__|__|__|__| **1=** Não tem data
47.	Se 44 é SIM, escrever o numero do cartão de seguimento da CRIANÇA<br>**1=** Outro (C.S.Manhiça) |__|__| /|__|__|__|/|__|__|/|__|__|__|__|__| **2=** Fora da Manhiça |__|__|__|__|__|__|__|__|__|__|__|__|__|__|
48.	Se 44 e NÃO, por quê?<br>**1=** Não tem<br>**2=** Perdeu<br>**3=** Recusa<br>**4=** Inacessível **(*voltar noutro dia)***<br>**5=** Outro |__|__|__|__|__|__|__|__|__|__|__|__|__|__|
	**RESULTADOS DO TESTE**
49.	***APRESENTAR A POSSIBILIDADE DE FAZER O TESTE PARA MAE E A CRIANÇA AGORA***<br>**A MÃE fez teste de HIV em casa dela agora?**<br>**1=** Sim<br>**2=** Não
50.	**Se 49 é SIM, resultado do teste de HIV da MÃE:**<br>**1=** Positivo<br>**2=** Negativo<br>**3=** Indeterminado
51.	**Se 49 é NÃO, porquê?**<br>**1=** HIV positiva em seguimento TARV (mostrou o cartão/ePTS)<br>**2=** HIV negativa com teste feito a menos de treis meses (mostrou cartão)<br>**3=** Obito (Mãe falecida)<br>**4=** Recusa<br>**5=** Recusa do parceiro ou familiares<br>**6=** Outro |__|__|__|__|__|__|__|__|__|__|__|__|__|__|
52.	**Amostra (DBS) da MÃE colhida?** **1=** Sim **2=** Não **3=** Não aplicavel
53.	**NIDANID MÃE** ☐
54.	**Se CRIANÇA é > 18 m, fez teste de HIV em casa dela agora?**<br>**1=** Sim<br>**2=** Não
55.	**Se 54 é SIM, resultado do teste de HIV da CRIANÇA:**<br>**1=** Positivo<br>**2=** Negativo<br>**3=** Indeterminado
56.	**Se 53 é NÃO, porquê?**<br>**1=** HIV positivo em seguimento TARV (mostrou o cartão/ePTS)<br>**2=** HIV negativo com teste feito a menos de tres meses (mostrou cartão)<br>**3=** Criança falecida<br>**4=** Recusa da mãe<br>**5=** Outro |__|__|__|__|__|__|__|__|__|__|__|__|__|__|
57.	**Amostra (DBS) da CRIANÇA colhida?** **1=** Sim **2=** Não **3=** Não aplicavel

58.	**NIDA CRIANÇA** [ ]
59.	**Se CRIANÇA é < 18 m, amostra DBS foi colhida na sua casa:**<br>**1=** Sim<br>**2=** Não
60.	**Se 59 é NÃO, porquê?**<br>**1=** HIV positivo em seguimento TARV (mostrou o cartão/ePTS)<br>**2=** Criança falecida<br>**3=** Recusa da mãe<br>**4=** Outro |__|__|__|__|__|__|__|__|__|__|__|
	**INFORMAÇÃO SOBRE A GRAVIDEZ DA CRIANÇA**
61.	**Durante a gravidez da CRIANÇA, a MÃE foi as consultas prenatais?**<br>**1=** Sim **2=** Não **3=** Não sabe
62.	**Antes da gravidez desta CRIANÇA, a MÃE sabia que ela era HIV positiva?**<br>**1=** Sim **2=** Não **3=** Não sabe
63.	***Se a MÃE refere NÂO conhecer o seu seroestado antes da gravidez desta CRIANÇA***<br>**Durante a gravidez, a MAE foi testada para HIV?**<br>**1=** Sim **2=** Não **3=** Não sabe
64.	**Quantas vezes a MAE fez teste de HIV durante a gravidez?**<br>**1=** 1 vezes<br>**2=** 2 vezes<br>**3=** > 2 vezes<br>**88=** Não sabe
65.	**Durante a <u>gravidez</u> ou o <u>parto</u>, qual foi o resultado do PRIMEIRO teste de HIV?**<br>**1=** Positivo<br>**2=** Negativo<br>**3=** Indeterminado<br>**88=** Não sabe
66.	**Durante a <u>gravidez</u> ou o <u>parto</u>, qual foi o resultado do ÚLTIMO/UNICO teste de HIV?**<br>**1=** Positivo<br>**2=** Negativo<br>**3=** Indeterminado<br>**88=** Não sabe
67.	**Durante a gravidez, a MAE tomou regularmente os comprimidos contra o HIV e ainda esta tomar?**<br>**1=** Sim **2=** Não **3=** Não sabe
68.	***Pedir ao participante para mostrar a documentação dela e da CRIANÇA referente a medicação***<br>**Que documentação apresentou? (*Multiresposta*)**<br>**1=** Ficha prenatal/caderneta da mulher<br>**2=** Cartão de seguimento nas consultas de HIV da MAE<br>**3=** Cartão da CRIANÇA<br>**4=** Cartão de seguimento nas consultas de HIV da CRIANÇA<br>**5=** Não tem<br>**6=** Perdeu<br>**7=** Inacessivel de momento<br>**8=** Recusa

	**9=** Outro |__|__|__|__|__|__|__|__|__|__|__|__|__|
69.	**Que tipo de comprimidos recebeu a MAE durante a gravidez?**<br>**1=** AZT e NVP+ Duovir em trabalho de parto e 7 dias apos o parto)<br>**2=** Opcao B+ Triple ARV<br>**3=** NVP dose única em trabalho de parto<br>**4=** Não sabe/ Impossivel de establecer<br>**5=** Cartao em mao estado ou com letras illegiveis<br>**6=** Outra |__|__|__|__|__|__|__|__|__|__|__|__|__|
70.	**Depois do nascimento, a MAE recebeu comprimidos contra HIV?**<br>**1=** Sim **2=** Não **3=** Não sabe
71.	**E a CRIANÇA, recebeu xarope para prevenir a infecção por HIV?**<br>**1=** Sim **2=** Não **3=** Não sabe
72.	**Depois do parto, que xarope tomou a CRIANÇA?**<br>**1=** Nevirapina<br>**2=** AZT<br>**3=** Não sabe/ impossível de estabelecer<br>**4=** Cartão em mão estado ou com letras ilegíveis<br>**5=** Outra |__|__|__|__|__|__|__|__|__|__|__|__|__|
73.	**Depois do parto, quanto tempo tomou a CRIANÇA os comprimidos?**<br>|__|__| **1=** Meses **2=** Anos **3=** Dias **4=** Não sabe
74.	**Alguma vez a criança recebeu uma transfusão de sangue?** **1=** Sim **2=** Não **3=** Não sabe
75.	**Se 74 é SIM, quantas |__|__|**
76.	**Se 74 é SIM, a quanto tempo foi a ultima?** |__|__| **1=** Meses **2=** Anos **88=** Não sabe
	**FIM**
77.	**Codigo conselheiro |__|__|__|**
78.	**Data da Visita** |__|__|- |__|__|__|-201|__|
