# Supplementary material for: Prompt HIV diagnosis and antiretroviral treatment in postpartum women is crucial for prevention of mother to child transmission during breastfeeding: Survey results in a high HIV prevalence community in southern Mozambique after the implementation of Option B+
Source: PLoS One. 2022 Aug 2;17(8):e0269835. doi: 10.1371/journal.pone.0269835 (PMC9345360; doi:10.1371/journal.pone.0269835)
Supplement: S2 Appendix — (ZIP) [file pone.0269835.s002.zip › SSP_METRO_001_A04_v02_PT.pdf]

	**INFORMAÇÃO SOCIO-DEMOGRÁFICA**	
1.	**Número de estudo do CUIDADOR METR - |__|__|__|__|**	
2.	**Idade do CUIDADOR em anos** |__|__| anos	
3.	**Agregado actual do CUIDADOR** |__|__|__|__|__| - |__|__|__|	
4.	**Sexo do CUIDADOR** **1=** Homem **2=** Mulher	
5.	**Qual é o parentesco do CUIDADOR com a MAE da CRIANÇA**<br>**1=** Pãe/mae<br>**2=** Avô<br>**3=** Irmã/Irmão<br>**4=** Vizinho<br>**5=** Tia/tio<br>**6=** Cunhada/cunhado<br>**7=** Nenhum<br>**8=** Outro |__|__|__|__|__|__|__|__|__|__|__|__|__|__|	
6.	**Qual é o parentesco do CUIDADOR com a CRIANÇA**<br>**1=** Pãe<br>**2=** Mãe adoptiva<br>**3=** Avô<br>**4=** Irmã<br>**5=** Irmão<br>**6=** Vizinho<br>**7=** Tio<br>**8=** Tia<br>**9=** Padrinho<br>**10=** Madrinha<br>**11=** Outro |__|__|__|__|__|__|__|__|__|__|__|__|__|__|	
7.	**Estado civil da MÃE:**<br>**1=** Solteiro (nunca viveu maritalmente)<br>**2=** Casado<br>**3=** União-de-facto<br>**4=** Divorciado<br>**5=** Separado<br>**6=** Viuvo	
8.	**Nível de escolaridade da MÃE:**<br>**1=** Analfabeto<br>**2=** Abaixo de 5 grau<br>**3=** 5 ª classe<br>**4=** 7 ª classe<br>**5=** 10 ª classe<br>**6=** 12 ª classe<br>**7=** Ensino técnico elementar	**8=** Ensino técnico básico<br>**9=** Ensino técnico médio<br>**10=** Bacharel<br>**11=** Licenciatura<br>**12=** Mestrado<br>**13=** Doutoramento<br>**14=** Outro |__|__|__|__|__|__|__|__|__|__| **88=** Não sabe

9.	**Principal material com que esta construida a casa onde que esta a dormir a CRIANCA?**<br>**1=** Blocos de cimento<br>**2=** Bloco de tijolo<br>**3=** Madeira/zinco<br>**4=** Bloco de adobe<br>**5=** Caniço/Pau/Bambu/Palmeira<br>**6=** Pau maticado<br>**7=** Lata/Carvão/Papel/Saco/Casca<br>**8=** Outros, especifique |__|__|__|__|__|__|__|__|__|__|__|__|
10.	**Principal fonte de agua de onde que bebem na casa da CRIANÇA:**<br>**1=** Agua emgarrafada<br>**2=** Agua canalizada dentro de casa<br>**3=** Água canalizada no quintal<br>**4=** Fontanário<br>**5=** Poço/ Furo protegido com bomba manual<br>**6=** Poço sem bomba<br>**7=** Água do Rio/Lago/Lagoa<br>**8=** Água da chuva<br>**9=** Outro |__|__|__|__|__|__|__|__|__|__|__|__|
11.	**Tipo de casa de banho que fazem servir na casa da CRIANÇA:**<br>**1=** Retrete ligada a fossa séptica<br>**2=** Latrina Melhorada<br>**3=** Latrina tradicional melhorada<br>**4=** Latrina não melhorada<br>**5=** Latrina compartida com um outro agregado<br>**6=** Não tem<br>**7=** Outro |__|__|__|__|__|__|__|__|__|__|__|__|
12.	**Qual é a principal fonte de rendimento do agregado onde vive a CRIANÇA?**<br>**1=** Camponês<br>**2=** Assalariado<br>**3=** Sem salario fixo<br>**4=** Não sabe ou não quere responder<br>**5=** Outro |__|__|__|__|__|__|__|__|__|__|__|__|
13.	**Religião da MAE:**<br>**1=** Católica<br>**2=** Protestante/Anglicana<br>**3=** Cristão indeterminado<br>**4=** Islâmica<br>**5=** Hindus<br>**6=** Zione/ Sião<br>**7=** Animistas<br>**8=** Envangelica / pentecostal<br>**9=** Ateus<br>**10=** Outro (especifique) |__|__|__|__|__|__|__|__|__|__|__|__| **88=** Não sabe<br>**99=** Recusa

<table>
<tr><td>14.</td><td>HISTORIA CLINICA DA MÃE</td></tr>
<tr><td></td><td>A MAE da criança tem mais filhos? 1= Sim 2= Não 3= Não sabe 4= Recusa</td></tr>
<tr><td>15.</td><td>Quantas crianças tem a MAE em total? |__|__| crianças
15.1 Queres testar ao resto das crianças menores de 48m? 1= Sim 2= Não 3= Não tem <48m</td></tr>
<tr><td>16.</td><td>Algumas das crianças da MAE que nasceram vivas morreram depois?
1= Sim 2= Não 3= Não sabe</td></tr>
<tr><td>17.</td><td>Preencher para cada uma das crianças mortas
Se 16 e SIM, idade da criança quando morreu |__|__| 1= Dias 2= Meses 3= Anos 4= Não sabe</td></tr>
<tr><td>18.</td><td>O CUIDADOR sabe se alguma vez a MÃE fez teste de HIV? 1= Sim 2= Não 3= Não sabe</td></tr>
<tr><td>19.</td><td>Pedir ao participante para mostrar a docuentação da MAE que acredite o teste (ficha prenatal/ ou cartão da criança/ ou cartão de seguimento do GATV)
O CUIDADOR mostrou alguma documentação? 1= Sim 2= Não</td></tr>
<tr><td>20.</td><td>Se 19 é SIM, que documentação apresentou? (Nota: Esta pergunta aceita multiplas opçoes)
1= Ficha prenatal/caderneta da mulher
2= Cartão da criança
3= Cartão de seguimento do GATV
4= Cartão de seguimento nas consultas de HIV
5= Outro |__|__|__|__|__|__|__|__|__|__|__|__|</td></tr>
<tr><td>21.</td><td>Se 19 é SIM, data do ÚLTIMO teste registado no documento |__|__|/|__|__|/|__|__|__|__|
888= Não tem data</td></tr>
<tr><td>22.</td><td>Se mostrou o cartao de seguimento, escrever o numero do cartão de seguimento da MÃE
1= Outro (C.S.Manhiça) |__|__| /|__|__|__|/|__|__|/|__|__|__|__|__|
2= Fora da Manhiça |__|__|__|__|__|__|__|__|__|__|__|__|__|</td></tr>
<tr><td>23.</td><td>Se 19 é NÃO, porquê?
1= Não tem
2= Perdeu
3= Inacessivel neste momento
4= Recusa
5= Outro |__|__|__|__|__|__|__|__|__|__|__|__|</td></tr>
<tr><td>24.</td><td>Se 19 é NÃO, o CUIDADOR sabe quando que a MÃE fez teste por ULTIMA vez?
|__|__| 1= Meses 2= Anos 3= Não sabe</td></tr>
<tr><td>25.</td><td>O CUIDADOR sabe se a MÃE era HIV positiva durante a gravidez da CRIANCA?
1= Sim 2= Não 3= Não sabe</td></tr>
<tr><td>26.</td><td>O CUIDADOR sabe se a MÃE foi diagnosticada apos o nascimento da CRIANCA?
1= Sim 2= Não 3= Não sabe</td></tr>
<tr><td>27.</td><td>O CUIDADOR sabe quando que a MÃE foi diagnosticada de HIV?
|__|__| 1= Meses 2= Anos 3= Não sabe</td></tr>
<tr><td>28.</td><td>CARACTERISTICAS DA CRIANÇA</td></tr>
<tr><td></td><td>Idade da CRIANÇA (preencher pelo conselheiro)
1= < 18 meses
2= > 18 meses</td></tr>
<tr><td>29.</td><td>Das crianças <u>biologicas</u> que teve a MAE, qual é a ordem de nascimento da CRIANÇA?
Colocar o numero de ordem (1 = Pimeira; 2= Segunda….) |__|__| 88= Não sabe</td></tr>
<tr><td>30.</td><td>A CRIANÇA foi nascida em Mozambique? 1= Sim 2= Não 3= Não sabe</td></tr>
</table>

31.	**Onde que a CRIANÇA nasceu?**<br>**1=** Centro de Saúde da Periferia<br>**2=** Hospital Distrital da Manhiça<br>**3=** Casa<br>**4=** Na casa do curandeiro<br>**5=** No caminho da unidade sanitaria<br>**6=** Recusa<br>**88=** Não sabe
32.	**A CRIANÇA amamentou nos últimos <u>dois meses</u>?** **1=** Sim **2=** Não
33.	**Quem foi a pessoa que amamentou a CRIANÇA?**<br>**1=** A mãe<br>**2=** O cuidador<br>**3=** Outro (escrever a relaçao com a criança) |__|__|__|__|__|__|__|__|__|__|__|
34.	**Se 32 é NÃO, a quanto tempo deixou de amamentar?**<br>|__|__| **1=** Meses **2=** Anos **3=** Não sabe
35.	**Alguma vez a CRIANÇA fez teste de HIV?** **1=** Sim **2=** Não **3=** Não sabe
36.	*O resto das perguntas, só se pergunta 35 é SIM*<br>**A que idade a CRIANÇA foi testada pela PRIMEIRA vez?**<br>**1=** < de dois meses de idade<br>**2=** 2 meses – 1 ano de idade<br>**3=** > de 1 ano de idade<br>**88=** Não sabe
37.	**Quantas vezes a CRIANÇA fez teste de HIV?**<br>**1=** 1 vezes<br>**2=** 2 vezes<br>**3=** > 2 vezes<br>**88=** Não sabe
38.	**Qual foi o resultado da CRIANÇA no PRIMEIRO/UNICO teste?**<br>**1=** Positivo<br>**2=** Negativo<br>**3=** Indeterminado<br>**88=** Não sabe
39.	**Qual foi o resultado da CRIANÇA no ÚLTIMO teste?**<br>**1=** Positivo<br>**2=** Negativo<br>**3=** Indeterminado<br>**4=** Nao tem feito mais testes<br>**88=** Não sabe
40.	**Alguma vez a CRIANÇA começo a tomar comprimidos/xarope contra o HIV?**<br>**1=** Sim **2=** Não **3=** Não sabe
41.	***Pedir ao participante para mostrar o cartão da CRIANÇA***<br>**O CUIDADOR mostrou alguma documentação da CRIANÇA?** 1= Sim 2= Não
42.	Se 41 é SIM, que documentação apresentou?<br>**1=** Ficha prenatal/caderneta da mulher<br>**2=** Cartão da criança

	**3=** Cartão de seguimento do GATV<br>**4=** Cartão de seguimento da CRIANÇA nas consultas de HIV<br>**5=** Outro |__|__|__|__|__|__|__|__|__|__|__|__|__|
43.	Se 41 é SIM, data do ÚLTIMO teste registado no documento |__|__|/|__|__|/|__|__|__|__| **1=** Não tem data
44.	Se 41 é SIM, escrever o numero do cartão de seguimento da CRIANÇA<br>**1=** Outro (C.S.Manhiça) |__|__| /|__|__|__|/|__|__|/|__|__|__|__|__|__| **2=** Fora da Manhiça |__|__|__|__|__|__|__|__|__|__|__|__|__|
45.	Se 41 e NÃO, porquê?<br>**1=** Não tem<br>**2=** Perdeu<br>**3=** Recusa<br>**4=** Inacessível **(*voltar noutro dia)***<br>**5=** Outro |__|__|__|__|__|__|__|__|__|__|__|__|__|
	**RESULTADOS DO TESTE**
46.	***APRESENTAR A POSSIBILIDADE DE FAZER O TESTE PARA CUIDADOR E A CRIANÇA AGORA***<br>**Se CRIANÇA é > 18 m, fez teste de HIV na sua casa:**<br>**1=** Sim<br>**2=** Não
47.	**Se 46 é SIM, resultado do teste de HIV da CRIANÇA:**<br>**1=** Positivo<br>**2=** Negativo<br>**3=** Indeterminado
48.	**Se 46 é NÃO, porquê?**<br>**1=** HIV positivo em seguimento TARV (mostrou o cartão/ePTS)<br>**2=** HIV negativo com teste feito a menos de treis meses (mostrou cartão)<br>**3=** Criança falecida<br>**4=** Recusa do criador<br>**5=** Outro |__|__|__|__|__|__|__|__|__|__|__|__|__|
49.	**Amostra (DBS) da CRIANÇA colhida?** **1=** Sim **2=** Não **3=** Não aplicavel
50.	**NIDA CRIANÇA** [ ]
51.	**Se CRIANÇA é < 18 m, amostra DBS foi colhida na sua casa:**<br>**1=** Sim<br>**2=** Não
52.	**Se 51 é NÃO, porquê?**<br>**1=** HIV positivo em seguimento TARV (mostrou o cartão/ePTS)<br>**2=** Criança falecida<br>**3=** Recusa do cuidador<br>**4=** Outro |__|__|__|__|__|__|__|__|__|__|__|__|__|
	**INFORMAÇÃO SOBRE A GRAVIDEZ DA CRIANÇA**
53.	**Durante a gravidez da CRIANÇA, a MÃE foi as consultas prenatais?**<br>**1=** Sim **2=** Não **3=** Não sabe
54.	**Antes da gravidez desta CRIANÇA, a MÃE sabia que ela era HIV positiva?**<br>**1=** Sim **2=** Não **3=** Não sabe

55.	***Se o CUIDADOR refere que a MAE NÂO conhecia o seu seroestado antes da gravidez da CRIANÇA***<br>**Durante a gravidez, a MAE foi testada para HIV?**<br>**1=** Sim **2=** Não **3=** Não sabe
56.	**Quantas vezes a MAE fez teste de HIV durante a gravidez?**<br>**1=** 1 vezes<br>**2=** 2 vezes<br>**3=** > 2 vezes<br>**88=** Não sabe
57.	**Durante a <u>gravidez</u> ou o <u>parto</u>, qual foi o resultado do PRIMEIRO teste de HIV?**<br>**1=** Positivo<br>**2=** Negativo<br>**3=** Indeterminado<br>**4=** **88=** Não sabe
58.	**Durante a <u>gravidez</u> ou o <u>parto</u>, qual foi o resultado do ÚLTIMO/UNICO teste de HIV?**<br>**1=** Positivo<br>**2=** Negativo<br>**3=** Indeterminado<br>**88=** Não sabe
59.	**Durante a gravidez, a MAE tomou comprimidos contra o HIV? Ou comprimidos para prevenir que a criança apanhara HIV?**<br>**1=** Sim **2=** Não **3=** Não sabe
60.	***Pedir ao participante para mostrar a documentação da MAE e da CRIANÇA referente a medicação***<br>**Que documentação apresentou? (*Multiresposta*)**<br>**1=** Ficha prenatal/caderneta da mulher<br>**2=** Cartão de seguimento nas consultas de HIV da MAE<br>**3=** Cartão da CRIANÇA<br>**4=** Cartão de seguimento nas consultas de HIV da CRIANÇA<br>**5=** Não tem<br>**6=** Perdeu<br>***7=*** Inacessivel de momento ***(voltar num outro dia)***<br>**8=** Recusa<br>**9=** Outro |__|__|__|__|__|__|__|__|__|__|__|__|
61.	**Que tipo de comprimidos recebeu a MAE durante a gravidez?**<br>**1=** AZT e NVP+ Duovir em trabalho de parto e 7 dias apos o parto)<br>**2=** Opção B+ Triple ARV<br>**3=** NVP dose única em trabalho de parto<br>**4=** Não sabe/ Impossível de estabelecer<br>**5=** Cartão em mão estado ou com letras ilegíveis<br>**6=** Outra |__|__|__|__|__|__|__|__|__|__|__|__|
62.	**Depois do nascimento, a MAE recebeu comprimidos contra o HIV?**<br>**1=** Sim **2=** Não **3=** Não sabe
63.	**E a CRIANÇA, recebeu xarope para prevenir a infecção?**<br>**1=** Sim **2=** Não **3=** Não sabe
64.	**Depois do parto, que comprimidos tomou a CRIANÇA?**<br>**1=** Nevirapina<br>**2=** AZT<br>**3=** Não sabe/ Impossível de estabelecer<br>**4=** Cartão em mão estado ou com letras ilegíveis

	**5=** Outra |__|__|__|__|__|__|__|__|__|__|__|__|__|__|
65.	**Depois do parto, quanto tempo tomou a CRIANÇA os comprimidos?**<br>|__|__| **1=** Meses **2=** Anos **3=** Dias **4=** Não sabe
66.	**Alguma vez a criança recebeu uma transfusão de sangue?** **1=** Sim **2=** Não **3=** Não sabe
67.	**Se 74 é SIM, quantas |__|__|**
68.	**Se 74 é SIM, a quanto tempo foi a ultima?** |__|__| **1=** Meses **2=** Anos **88=** Não sabe
	**FIM**
69.	**Codigo conselheiro |__|__|__|**
70.	Data da visita |__|__|- |__|__|__|-201|__|
