# Supplementary material for: Prompt HIV diagnosis and antiretroviral treatment in postpartum women is crucial for prevention of mother to child transmission during breastfeeding: Survey results in a high HIV prevalence community in southern Mozambique after the implementation of Option B+
Source: PLoS One. 2022 Aug 2;17(8):e0269835. doi: 10.1371/journal.pone.0269835 (PMC9345360; doi:10.1371/journal.pone.0269835)
Supplement: S2 Appendix — (ZIP) [file pone.0269835.s002.zip › SSP_METRO_001_A05_v01_PT.pdf]

**SSP_METRO_001_A05_v01_PT**		**Visita de recrutamento no campo**	
cism centro de investigação em saúde de manhiça	**Estudo: METRO**<br>**Inquérito: CRF testado crianças**		Serial Number

	**RESULTADOS DO RESTO DAS CRIANÇAS DA CASA**
1.	**Numero de estudo da MAE/CUIDADOR** **METR - |__|__|__|__|**
2.	**Quantas crianças menores de 48 meses há no agregado?** |__|__|
3.	**Quantas crianças foram testadas?** |__|__|
4.	***Repetir para cada criança NAO testada***<br>**Porque a criança não foi testada?**<br>**1=** Recusa da mãe/cuidador<br>**2=** Recusa de uma familiar<br>**3=** Criança ausente<br>**4=** A criança é HIV positiva conhecida (mostrou cartão)<br>**5=** Criança HIV negativa com teste feito a menos de 3 meses (amostrou o cartão)<br>**6=** Outro |__|__|__|__|__|__|__|__|__|__|__|
5.	***Repetir para cada criança > 18 meses testada***<br>**Idade em meses** |__|__| meses
6.	**Resultado do teste da criança?**<br>**1=** Positivo<br>**2=** Negativo<br>**3=** Indeterminado
7.	***Repetir para cada criança < 18 meses testada***<br>**CHILD - |__|__|__|__|**
8.	**Perm ID da criança** |__|__|__|__| - |__|__|__| - |__|__| 88= Não tem
9.	**Nome da criança** |__|__|__|__|__|__|__|__|__|__|__|
10.	**Idade em meses** |__|__| meses
11.	**Sexo da criança** **1= Homem** **2=Mulher**
12.	**Foi colhida amostra de sangue (DBS)?**<br>**1=** Sim **2=** Não **3=** Não aplicável
13.	**NIDA CHILD** [ ]
	**FIM**
14.	Código conselheiro |__|__|__|
15.	Data da visita |__|__|- |__|__|__|-201|__|
