# Supplementary material for: Prompt HIV diagnosis and antiretroviral treatment in postpartum women is crucial for prevention of mother to child transmission during breastfeeding: Survey results in a high HIV prevalence community in southern Mozambique after the implementation of Option B+
Source: PLoS One. 2022 Aug 2;17(8):e0269835. doi: 10.1371/journal.pone.0269835 (PMC9345360; doi:10.1371/journal.pone.0269835)
Supplement: Attachment — Submitted filename: Comments on Prolonged breastfeeding is safe in HIV.docx [file pone.0269835.s003.docx]

**Prolonged breastfeeding is safe in HIV-exposed children: Survey results in a high HIV prevalence community in southern Mozambique after implementation of Option B+**

***Fernandez-Luis et al.***

This study looks at both duration of breastfeeding among HIV-exposed and HIV-unexposed infants as well as factors associated with postpartum transmission of HIV in Mozambique. HIV-exposed infants received six weeks of nevirapine, whether they were breastfeeding or not.

The authors conclude that women living with HIV breastfeed for a shorter median duration than women not living with HIV and that infants not receiving 6 weeks of nevirapine prophylaxis are at higher risk of HIV acquisition.

1) The study design is a bit confusing: women were chosen randomly to be interviewed but this was primarily a retrospective chart review combined with mother’s memories of what she did during pregnancy and postpartum.
2) What information do the authors have about maternal adherence to ART? In the era of U=U (undetectable equals untransmissible) in the world of sexual transmission, many obstetricians and pediatricians seek information on the effect of maternal adherence on transmission. Flynn et al have documented a 0.3% transmission rate at 6 months and 0.6% transmission rate at 12 months among women with undetectable viral loads. The Mozambique study addresses the “real world” of not having easy availability of viral loads but there is little said about maternal adherence. The authors cite a study by Pfeiffer 2017 that documented only 38% of women living with HIV still obtaining ART refills for themselves 90 days postpartum but do not comment on adherence among their study participants. Did their survey ask about adherence?
3) The actual risk of transmission appears to be 51% of 5.3%, or 2.7% (per Figure 1). That rate is significantly higher than the transmission rates in the PROMISE study (Flynn et al). Please comment.
4) I would also like some discussion of why the authors think that women living with HIV breastfed for a shorter period of time than women who did not have HIV. I can imagine that those with HIV may have been aware of some risk of transmission of HIV via breastmilk and, therefore, weaned their babies earlier. Did the surveys ask women why they stopped breastfeeding when they did? If not, what reasons do the authors think would explain early cessation?
5) If the standard of care in Mozambique is to give six weeks of infant prophylaxis, what were the reasons mothers gave for not giving it?
6) Of special interest to us in the U.S., Canada, and Europe, adding infant prophylaxis in this study was associated with a significant decrease in postpartum transmission. In high resource countries some pediatricians have opted to give nevirapine through cessation of breastfeeding in addition to continuing maternal ART. This study lends credence to that approach.

Although this study attempts to add important information to the literature, I am not sure that the study design allows us to confidently draw conclusions from it.
